# CD93 overexpresses in liver hepatocellular carcinoma and represents a potential immunotherapy target

**DOI:** 10.3389/fimmu.2023.1158360

**Published:** 2023-07-07

**Authors:** Qianwei Jiang, Jing Kuai, Zhongyi Jiang, Weitao Que, Pusen Wang, Wenxin Huang, Wei Ding, Lin Zhong

**Affiliations:** ^1^ Department of General Surgery, Shanghai General Hospital, Shanghai Jiao Tong University School of Medicine, Shanghai, China; ^2^ Department of Hepatobiliary Surgery, Weifang People’s Hospital, Shandong, Weifang, Shandong, China

**Keywords:** CD93, liver hepatocellular carcinoma, biomarker, immunotherapy target, immune infiltration

## Abstract

**Background:**

Liver hepatocellular carcinoma (LIHC) is one of the malignant tumors with high incidence as well as high death, which is ranked as the sixth most common tumor and the third highest mortality worldwide. CD93, a transmembrane protein, has been widely reported to play an important role in different types of diseases, including many types of cancer by mainly functioning in extracellular matrix formation and vascular maturation. However, there are few researches focusing on the role and potential function of CD93 in LIHC.

**Methods:**

In this study, we comprehensively analyzed the relationship between CD93 and LIHC. We not only discovered transcriptional expression of CD93 in LIHC by using the TIMER, GEPIA and UALCAN database, but also performed WB and IHC to verify the protein expression of CD93 in LIHC. Meantime, Kaplan-Meier Plotter Database Analysis were used to assess the prognosis of CD93 in LIHC. After knowing close correlation between CD93 expression and LIHC, there were STRING, GeneMania and GO and KEGG enrichment analyses to find how CD93 functions in LIHC. We further applied CIBERSORT Algorithm to explore the correlation between CD93 and immune cells and evaluate prognostic value of CD93 based on them in LIHC patients.

**Results:**

The transcriptional and protein expression of CD93 were both obviously increased in LIHC by above methods. There was also a significant and close correlation between the expression of CD93 and the prognosis of LIHC patients by using Kaplan-Meier Analysis, which showed that LIHC patients with elevated expression of CD93 were associated with a predicted poor prognosis. We found that the functions of CD93 in different cancers are mainly related to Insulin like growth factor binding protein 7 Gene (IGFBP7)/CD93 pathway via STRING, GeneMania and functional enrichment analyses. Further, our data obtained from CIBERSORT Algorithm suggested CD93 was also associated with the immune response. There is a close positive correlation between CD93 expression and the infiltration levels of all six types of immune cells (B cells, CD8+ T cells, CD4+ T cells, macrophages, neutrophils, and dendritic cells). Importantly, CD93 can affect the prognosis of patients with LIHC partially due to immune infiltration.

**Conclusion:**

Our results demonstrated CD93 may be a candidate predictor of clinical prognosis and immunotherapy response in LIHC.

## 1 Introduction

Liver hepatocellular carcinoma (LIHC), the major subtype of liver cancer, is ranked as the sixth most common tumor and the third highest mortality among all malignancies, whose causes mainly arise from chronic liver disease and chronic hepatitis B and C viral infection (1, 2). There are a variety of mature and effective diagnosis and treatments for LIHC, including surveillance with imaging technology and α-fetoprotein plasma levels every 6 months and hepatic resection, liver transplantation, and transarterial chemoembolization (3–6). But most patients are usually diagnosed as advanced liver cancer because of insidious onset, so that they can’t benefit from those treatments (7). In recent years, immunotherapy represented by immune checkpoint inhibitors has made huge and amazing breakthroughs to benefit more and more LIHC patients, particularly those with advanced cancer (8–10). However, there are still no clear and effective biomarkers to predict efficacy of immunotherapy for LIHC. Here, we found a potential gene, called CD93, to probably assume this important role.

CD93 is a transmembrane protein expressed in stem cells, monocytes, and endothelial cells, which consists of several domains, such as an extracellular domain with a C-type lectin domain (11–13). There are numerous studies that have found that CD93 plays a critical role in many diseases, including allergic asthma, diabetic wound healing, and many types of cancer (14–16). It’s worth noting that CD93 is involved in angiogenesis in human primary tumors. The interaction between CD93 and its specific ligand, Multimerin 2 (MMRN2), can contribute to endothelial cell adhesion and migration, thus promoting pathological angiogenesis (17–19). CD93 can also function in vascular maturation and extracellular matrix formation by boosting β1 integrin activation and fibronectin to promote angiogenesis (20). Furthermore, CD93 can play a critical role in innate immunity (21). Recent clinical studies have shown that high CD93 expression had a close relationship with the poor effects of immunotherapy (22, 23). In addition, the blockage of the IGFBP7/CD93 pathway brings an extensive increase of effector T cells, making tumors sensitive to immune checkpoint therapy (24, 25).

Although CD93 has been wildly reported to play a critical role in many types of cancer, there are few studies to reveal the value of CD93 in LIHC. The aim of this study was to explore the promising predictive value of CD93 for LIHC prognosis and immunotherapy.

## 2 Materials and methods

### 2.1 Tumor samples and collection

Human LIHC tissues and paired normal tissues were recruited in Shanghai General Hospital from January 2016 to January 2021. All patients with LIHC underwent surgery for the first time and had not previously received radiotherapy or chemotherapy. Written informed consent was obtained from each patient. This study was approved by the ethics committee of Shanghai General Hospital (2021KSQ341).

### 2.2 Western blotting

Protein extracted from tissues was using RIPA buffer (Beyotime, Shanghai, China) mixed with PMSF (Beyotime, Shanghai, China) for 30 min on ice, and then centrifuged at 12,000 rpm for 10 min at 4°C. Protein lysates were separated by using SDS-PAGE and transferred to PVDF membranes. After incubating with 5% Bovine serum albumin (BSA) for 1 h at room temperature (RT), the membranes were incubated with primary antibodies (anti-CD93 antibody, sc-365172, Santa Cruz, USA; anti- anti-β-actin antibody, Sangon Biotech, Shanghai, China) overnight at 4°C, washed with Tris Buffered Saline with Tween®20 (TBST) for 3 times, and further incubated with secondary antibodies (Sangon Biotech, Shanghai, China) for 1 h at RT, and developed using ECL solutions (Beyotime).

### 2.3 Immunohistochemistry staining and immunofluorescence staining

The samples were fixed in 10% paraformaldehyde for 24h and then embedded in paraffin wax. After deparaffinized and rehydrated, 5 µm thick slides were stained with hematoxylin & eosin (H&E) or primary antibodies (anti-CD93 antibody, sc-365172, Santa Cruz, USA), followed by incubation with horseradish peroxidase (HRP)-conjugated secondary antibody (Sangon, Shanghai, China). All the sections were observed using an AX-80 microscope (Olympus, Tokyo, Japan). The cells which were stained brown were considered positive (tumor tissue, *n* = 6; adjacent tissue, *n* = 6).

The samples were formalin-fixed and embedded in paraffin, then were deparaffinized, rehydrated, permeabilized, and rinsed. After those, we performed antigen repair in citrate buffer for 15 min and carried out the blocking in 5% BSA for 1 hour at room temperature. Then sections were stained with anti-CD93 (sc-365172, Santa Cruz, USA) anti-CD31/PECAM-1 (sc-18916, Santa Cruz, USA) and anti-IGFBP7 (171085, Abcam, USA) antibodies overnight at 4°C in a humidified box. Then, sections were incubated with secondary antibodies for 1 hour at RT and protected from light. After stain with DAPI, microscope images were taken of the sections.

### 2.4 Real-time PCR

Total RNA was extracted from human samples by using an Isolation Kit following the manufacturer’s instructions. Then we performed the reverse transcription by using the reverse transcription kit. After those, Real-time PCR was carried out with the SYBR qPCR Master Mix kit. The primer sequences used for gene analysis were as follows: CD93-Forward: 5’-GCCCCAGAATGCGGCAGACA-3’, CD93-Reverse: 5’-GCAGTCTGTCCCAGGTGTCGGA-3’; β-actin-Forward: 5’-AGGATTCCTATGTGGGCGAC-3’, β-actin-Reverse: 5’-ATAGCACAGCCTGGATAGCAA-3’.

### 2.5 Tumor immune estimation resource

TIMER (https://cistrome.shinyapps.io/timer/) is a comprehensive resource for performing systematical analysis of immune infiltrates across diverse cancer types (26). In this study, it was applied to evaluate the correlation between CD93 expression and the infiltration of immune cells and investigate the relationship between CD93 expression and different gene marker sets of immune cells.

### 2.6 Gene expression profiling interactive analysis

GEPIA (http://gepia.cancer-pku.cn/index.html) is a user-friendly web portal for gene expression analysis based on TCGA and GTEx data (27). In this study, it was used to explore the expression in HCC and clarify the relationships between CD93 and PD-1, PD-L1, CTLA-4, and VEGFA.

### 2.7 UALCAN

UALCAN (http://ualcan.path.uab.edu/) is a web-based tool for providing in-depth analyses of transcriptome data from The Cancer Genome Atlas (TCGA) and MET500 data (28). In this study, it is built to analyze the mRNA expression levels of CD93 in LIHC and the relationship between CD93 expression and patients’ gender, individual cancer stages, and pathological grades.

### 2.8 Kaplan-Meier plotter database analysis

KM Plotter (http://kmplot.com) is an online database that contains gene expression data and survival information. In this study, it is used to analyze the prognostic value of CD93 in LIHC, including overall survival (OS), progression-free survival (PFS) and disease-free survival (DSS) with hazard ratios (HRs) with 95% confidence intervals (95% CIs) and log-rank p-values.

### 2.9 GeneMANIA

GeneMANIA (http://www.genemania.org) is a flexible, user-friendly tool for generating hypotheses about gene function (29). In this study, it is applied to construct the gene-gene interaction network.

### 2.10 STRING

STRING (https://string-db.org/) is an online database for searching known protein interaction relationships. In this study, it is used to collect, score, and integrate all publicly available sources of protein-protein interaction (PPI) data, and to complement these with computational predictions of potential functions.

### 2.11 Gene ontology term and Kyoto encyclopedia of genes and genomes pathway enrichment analysis and gene set enrichment analysis

GO and KEGG analyses were applied to explore the biological functions of CD93 in LIHC. In this study, GO analysis is a powerful bioinformatics tool to determine the biological processes (BPs), cellular components (CCs) and molecular functions (MFs) related to CD93. GSEA was used to investigate the potential mechanisms of CD93.

### 2.12 Immune cell infiltration with the CIBERSORT algorithm

CIBERSORT (https://cibersort.stanford.edu/), is an established computational resource for characterizing the immune cell composition based on a validated leukocyte gene signature matrix containing 547 genes and 22 human immune cell subpopulations (30). In this study, it is applied to examine the correlations between CD93 expression and the immune cell subpopulation.

### 2.13 Statistical analysis

All statistical analyses were performed using R software 4.0.1. The results of Kaplan-Meier plots and GEPIA are displayed with HR and P or Cox P-values from a log-rank test. The correlation of CD93 gene expression was explored by Spearman’s correlation and statistical significance. The heat map of the correlations was generated by the R software package pheatmap with Spearman’s correlation. The P-values < 0.05 were considered statistically significant for all statistical analyses.

## 3 Results

### 3.1 Pan-cancer analysis of CD93 expression

**Figure 1:**
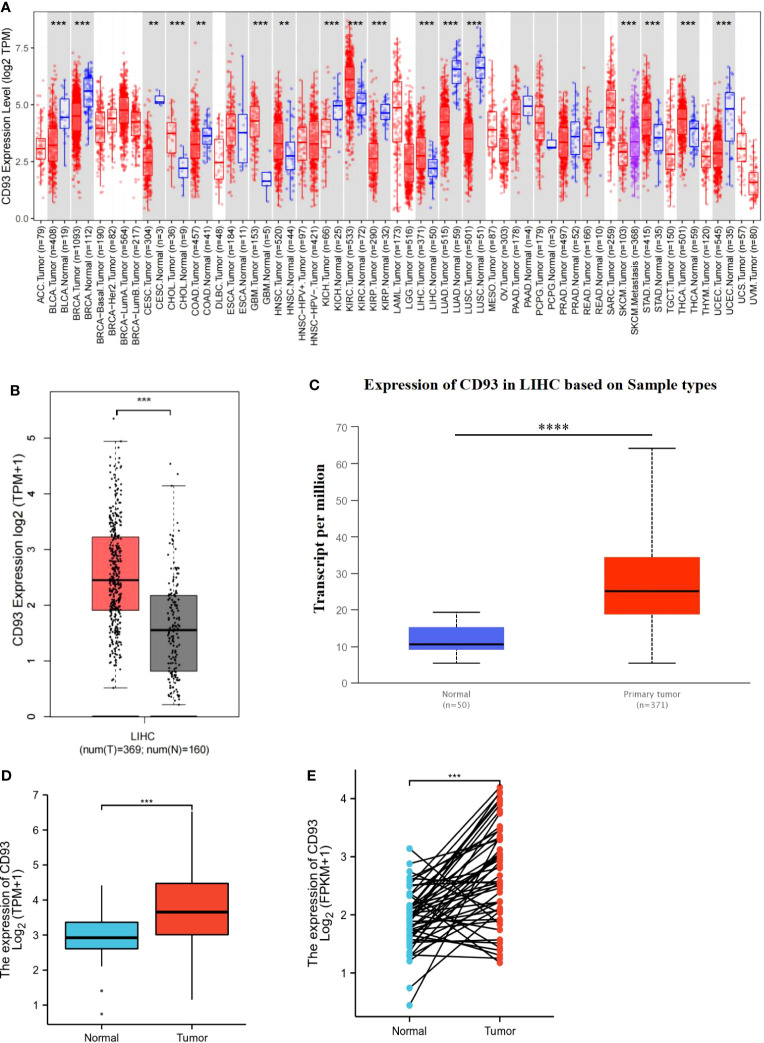
Transcriptional expression of CD93 in LIHC. **(A)** CD93 expression in different types of cancers was examined by using the TIMER database. **(B)** Increased expression of CD93 in LIHC compared to normal tissues in the GEPIA database. **(C)** Increased expression of CD93 in LIHC compared to normal tissues in the UALCAN database. **(D)** The expression of CD93 was higher in LIHC tissues by using the TCGA database. **(E)** CD93 was found to be highly expressed in LIHC tissues in 50 pairs of tumor tissues and paired adjacent tissues in the TCGA database. **p < 0.01, ***p < 0.001, ****p < 0.0001.

To investigate the mRNA expression of CD93 in tumor and normal tissues, we utilized an online tool, Tumor Immune Estimation Resource (TIMER), to find that the expression of CD93 between various tumors and adjacent tissues was tremendously different (**Figure 1A**). Compared with the normal tissues, higher expression of CD93 was observed in Bladder urothelial carcinoma (BLCA), Breast invasive carcinoma (BRCA), Cervical squamous cell carcinoma, and endocervical adenocarcinoma (CESC), Cholangiocarcinoma (CHOL), Colon adenocarcinoma (COAD), Kidney Chromophobe (KICH), Kidney renal papillary cell carcinoma (KIRP), Liver hepatocellular carcinoma (LIHC), Lung adenocarcinoma (LUAD), Lung squamous cell carcinoma (LUSC), Stomach adenocarcinoma (STAD), and Uterine Corpus Endometrial Carcinoma (UCEC). Similarly, we confirmed that higher expression of CD93 in LIHC than in normal tissues from the gene expression profiling interactive analysis (GEPIA), the UALCAN databases, and The Cancer Genome Atlas (TCGA) and Gene Expression Omnibus (GEO), respectively (**Figures 1B–D**; **Supplementary Figure 1A**). Furthermore, there was a significantly increased mRNA expression of CD93 in 50 paired LIHC tissues compared to paired adjacent normal tissues (**Figure 1E**). Then, we performed the Real-time PCR to verify the above conclusion (**Supplementary Figure 1B**). These findings suggest that CD93 expression is increased in LIHC patients and that it may play a pivotal role in the occurrence and progression of LIHC.

**Figure 2:**
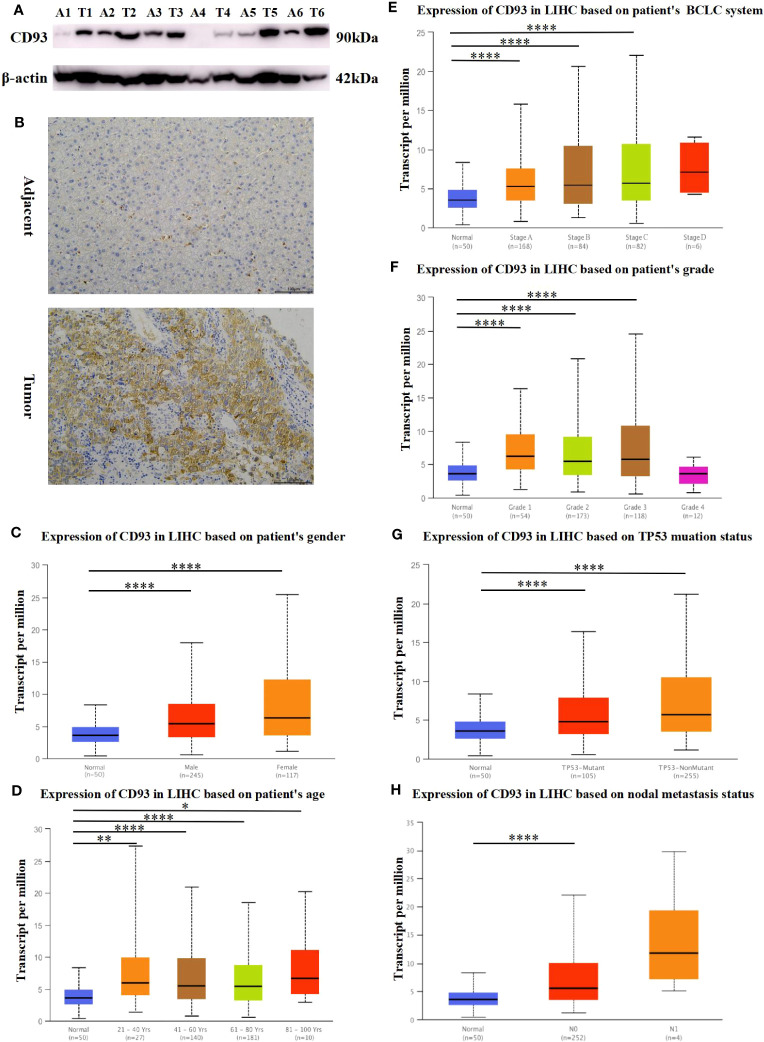
Protein expression of CD93. **(A)** Increased expression of CD93 in LIHC compared to normal tissues by WB. **(B)** Increased expression of CD93 in tumors compared to normal tissues by IHC.Box plots evaluating CD93 expression among different groups of patients based on clinical parameters using the UALCAN database. **(C)** gender, **(D)** age, **(E)** BCLC system, **(F)** Edmondson’s pathological grade, **(G)** TP53 mutation status, **(H)** nodal metastasis status. *p < 0.05, **p < 0.01, ***p < 0.001, ****p < 0.0001.

We also investigated the protein expression of CD93 in LIHC tumor tissues and adjacent tissues. The results of Western Blot and Real-time PCR have demonstrated that CD93 was markedly upregulated in LIHC tumor tissues than in adjacent tissues (**Figure 2A**). Not coincidentally, immunohistochemistry and immunofluorescence staining of LIHC tumor tissue and adjacent tissues also demonstrated the same results (**Figure 2B**; **Supplementary Figure 2**).

### 3.2 CD93 expression and clinical parameters of LIHC

Since the expression of CD93 increased significantly in patients with LIHC, we further explored the relationships between CD93 expression levels and clinical outcomes according to different clinical parameters by using the UALCAN database. As shown in **Figures 2C, D**, there were significant differences in CD93 expression in tumors and normal tissues of LIHC patients by gender and age group. Based on the BCLC system (31), CD93 expression was higher in patients with LIHC classified as stages A, B, and C, which suggested that there was a close correlation between CD93 expression and tumor progression (**Figure 2E**). Regarding Edmondson’s pathological grade of LIHC, a significant increase in CD93 expression was observed in LIHC patients in grades 1, 2, and 3 (**Figure 2F**). We then investigated the expression of CD93 in LIHC based on TP53 mutation status and found that the expression of CD93 in tumor tissues was higher than that in normal liver tissues regardless of TP53 mutation (**Figure 2G**). What’s more, upregulated CD93 expression was observed in LIHC patients with nodal metastases (**Figure 2H**). These results suggest that the expression level of CD93 is closely related to the clinical progression of LIHC.

### 3.3 Elevated expression of CD93 indicates poor prognosis for LIHC

**Figure 3:**
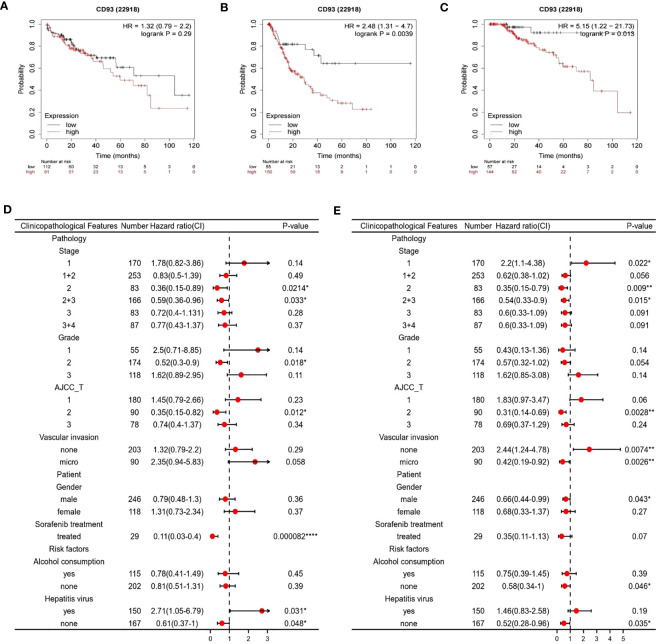
Survival curve evaluating the prognostic value of CD93. Survival curves using the Kaplan-Meier plotter are shown for **(A)** OS, **(B)** PFS, **(C)** DFS; A forest plot from the Kaplan-Meier database shows the correlation between CD93 expression and LIHC patients’ clinicopathological parameters, such as **(D)** OS, **(E)** PFS. *p < 0.05, **p < 0.01, ****p < 0.0001.

Based on our findings, we then examined the prognostic value of the CD93 gene by using the Kaplan Meier plotter database. According to the median expression of CD93, patients in the database were divided into high and low-expression subgroups. The results have shown that LIHC patients with higher expression of the CD93 gene who had no vascular invasion exhibited poor progression-free survival (PFS) (**Figure 3B**) and Disease-free survival (DFS) (**Figure 3C**), although there was no statistical difference in overall survival (OS) (**Figure 3A**). Moreover, we validated the prognostic value of CD93 according to various clinicopathological features using the Kaplan-Meier database. We found that high CD93 expression was significantly associated with poor OS in patients infected with the hepatitis virus, and with poor PFS when vascular invaded (**Figures 3D, E**). These results imply that CD93 expression possesses prognostic value in LIHC.

### 3.4 Identification of CD93 potential mechanism in LIHC

**Figure 4:**
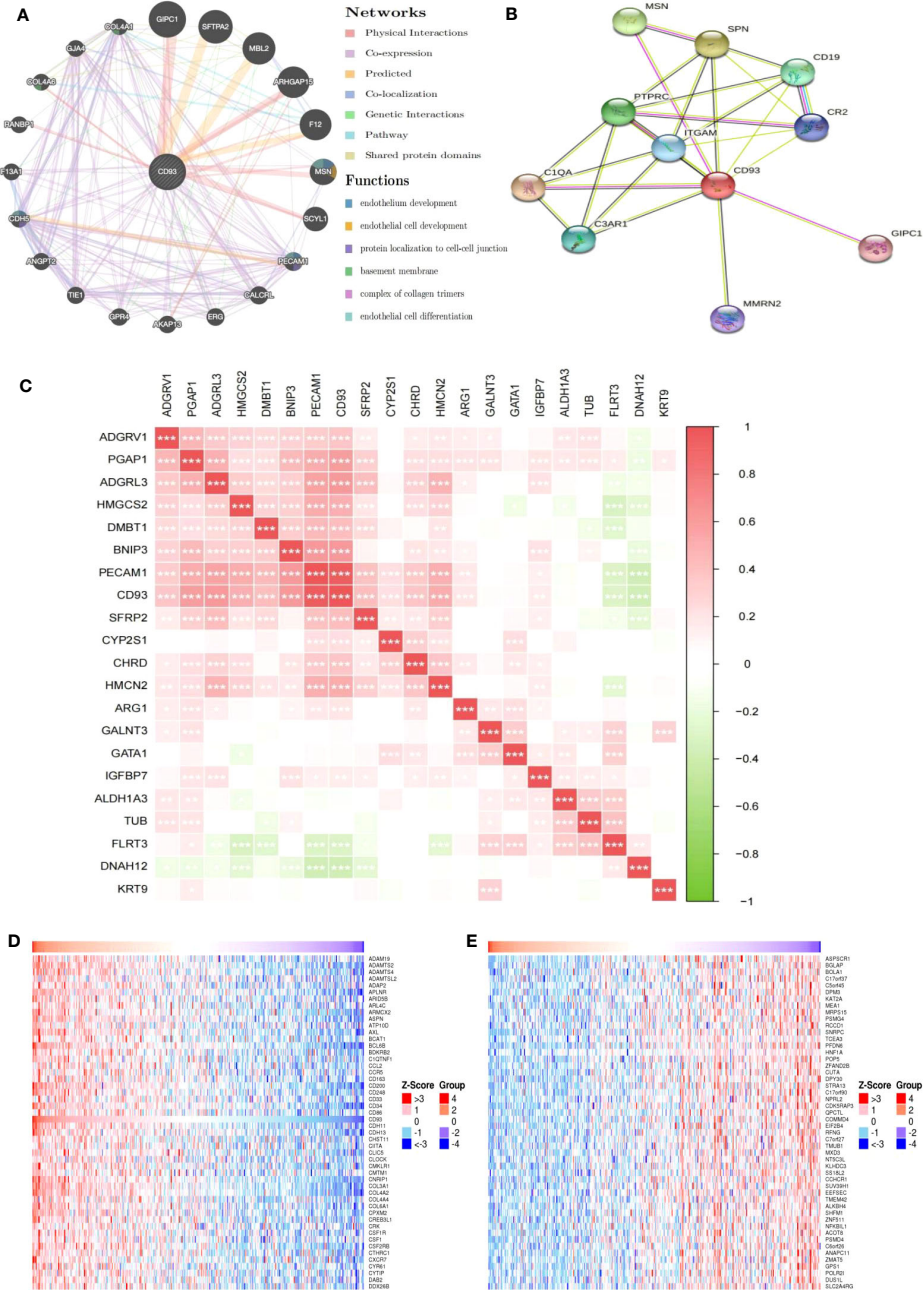
Genes and pathways closely related to CD93. **(A)** The gene-gene interaction network of CD93 was constructed using GeneMania. **(B)** The PPI network of CD93 was generated using STRING. **(C)** Heat maps showing the correlations between CD93 and other genes in LIHC. **(D)** Heat maps showing the top 50 genes positively correlated with CD93 in LIHC. **(E)** Heat maps showing the top 50 genes negatively correlated with CD93 in LIHC. *p < 0.05, **p < 0.01, ***p < 0.001.

In addition to elucidating the prognostic value of CD93 expression in LIHC, we also focused on the potential mechanisms involved in CD93 in LIHC. We generated the gene-gene interaction network to explore the altered neighboring genes of CD93 via GeneMania (**Figure 4A**). The results showed that the 20 most frequently altered genes were closely correlated with CD93, including A-kinase anchor protein 13 (AKAP 13) and Collagen alpha-1(IV) chain (COL4A1). Functional analysis revealed that these genes were significantly associated with endothelium development and others. We also produced the protein-protein interaction (PPI) network of CD93 through the STRING database and obtained 48 edges and 11 nodes, which included PDZ domain-containing protein GIPC1 and Complement C1q subcomponent subunit A (C1QA) (**Figure 4B**). Further, we investigated the correlation between CD93 and endothelial cell function-related genes based on the TCGA database (**Figure 4C**). As result, CD93 was positively and significantly correlated with Insulin-like growth factor-binding protein 7 (IGFBP7), post-GPI attachment to proteins inositol deacylase 1(PGAP1), and platelet and endothelial cell adhesion molecule 1 (PECAM1) whereas negatively correlated with fibronectin leucine rich transmembrane protein 3 (FLRT3), and dynein axonemal heavy chain 12 (DNAH12). In addition, we found that CD93 was correlated with vascular endothelial growth factor a (VEGFA) using the GEPIA and TIMER databases (**Supplementary Figures 3A, B**). We also performed IF staining to confirm the close contact between CD93 and IGFBP7 (**Supplementary Figure 3C**).

**Figure 5:**
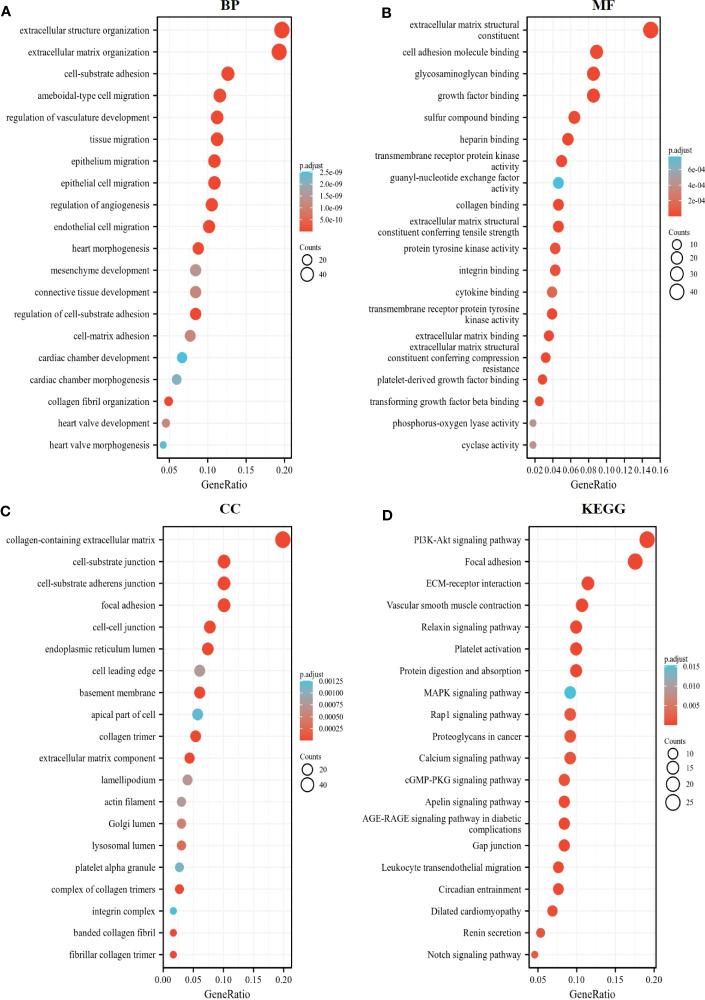
Enrichment analysis of CD93. The top 20 significant terms by GO and KEGG functional enrichment analysis showed in **(A)** BP, **(B)** MF, **(C)** CC, and **(D)** KEGG.

We further exploited the TCGA database to identify genes positively or negatively co-expressed with CD93. The top 50 genes that were positively and negatively correlated with CD93 in LIHC were shown in **Figures 4D**, **E**. To establish a clearer understanding of the biological functions and potential mechanisms involved in CD93 in the development of LIHC, we presented the top 20 significant terms by GO and KEGG functional enrichment analysis. As shown in the BP category, CD93 was enriched in the extracellular matrix organization, regulation of vasculature development, and regulation of angiogenesis (**Figure 5A**). Correspondingly, the enriched processes of MF were extracellular matrix structural constituent, cell adhesion molecule binding, and growth factor binding (**Figure 5B**), while the main enrichment of CC included collagen-containing extracellular matrix, cell-substrate junction, and collagen trimer (**Figure 5C**). Signaling pathway enrichment analysis demonstrated that high CD93 expression in LIHC was associated with the PI3K-Akt signaling pathway, ECM-receptor interaction, MAPK signaling pathway, etc. (**Figure 5D**).

### 3.5 Correlation analysis between CD93 expression and infiltrating immune cells

**Figure 6:**
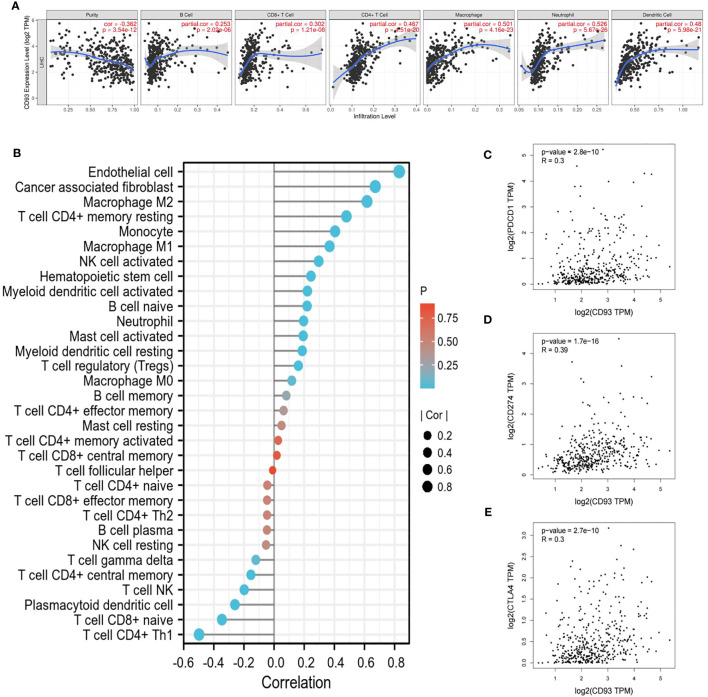
Relationship between CD93 and immune cells. **(A)** CD93 significantly associated with tumor purity and positively correlated with the infiltration of different immune cells using the TIMER database. **(B)** CD93 expression significantly correlated with the infiltration of immune cells in LIHC by using the CIBERSORT algorithm. **(C–E)** Scatterplots of the correlations between CD93 expression and **(C)** PD-1, **(D)** PD-L1 and **(E)** CTLA-4 in LIHC using the GEPIA database.

Given the complex hepatic immune microenvironment, we further evaluated the effect of CD93 expression in association with immune infiltrating cells on the occurrence and progression of LIHC using the TIMER database. We initially found a significant positive correlation between CD93 expression levels and the infiltration of six types of immune cells, including B cell, CD8^+^ T cell, CD4^+^ T cell, macrophage, neutrophil, and dendritic cell (**Figure 6A**). Furthermore, we estimated the associations of immune infiltration levels of immune cell subtypes with CD93 expression. As we showed, CD93 was notably positively correlated with the infiltration levels of endothelial cells, cancer-associated fibroblasts, M1/M2 macrophages, and activated Natural killer (NK) cells, whereas negatively correlated with the infiltration levels of Type 1 T helper cells, γδ T cells, central memory CD4^+^ T cells (**Figure 6B**).

### 3.6 Correlation between CD93 expression and diverse immune markers

To gain insight into the interaction between CD93 and immune responses, we utilized the TIMER database to verify the correlation between CD93 expression and various immune features in LIHC, including B cells, T cells, CD8^+^ T cells, monocytes, tumor-associated macrophages (TAMs), M1/M2 macrophages, neutrophils, NK cells, and dendritic cells (**Table 1**). Based on calibrated tumor purity, we confirmed that CD93 expression correlated significantly with most of the representative markers in a variety of immune cells in LIHC (**Table 1**).

**Table 1:** Correlation analysis between CD93 and gene markers of immune cells in TIMER.

Description	Gene markers	LIHC			
		None		Purity	
		Cor	P	Cor	P
**B cell**	CD19	0.214692912	****	0.112166065	*
	CD79A	0.306124833	****	0.17127049	**
**T cell (general)**	CD3D	0.212227139	****	0.083895849	0.1199
	CD3E	0.398034483	****	0.277357047	****
	CD2	0.352582267	****	0.233499214	****
**CD8+ T cell**	CD8A	0.343579524	****	0.249149122	****
	CD8B	0.197541036	***	0.087212881	0.1059
**Monocyte**	CD86	0.548714213	****	0.480956881	****
	CSF1R	0.564914449	****	0.491071275	****
**TAM**	CCL2	0.568209815	****	0.484845407	****
	CD68	0.420702313	****	0.325439064	****
	IL10	0.430418027	****	0.324665302	****
**M1**	IRF5	0.348446454	****	0.377669482	****
	PTGS2	0.649824421	****	0.584545518	****
	NOS2	0.437941659	****	0.431980104	****
**M2**	CD163	0.565593305	****	0.502327393	****
	VSIG4	0.537297344	****	0.464709792	****
	MS4A4A	0.572326731	****	0.505990943	****
**Neutrophils**	CEACAM8	0.021500083	0.6798	-0.018706305	0.7292
	ITGAM	0.475869313	****	0.415199851	****
	CCR7	0.479802725	****	0.361253566	****
**Natural killer cell**	KIR2DL1	0.082681125	0.1119	0.075846689	0.1598
	KIR2DL3	0.176757483	***	0.131086101	*
	KIR2DL4	0.103274489	*	0.063190688	0.2417
	KIR3DL1	0.207017402	****	0.205497668	***
	KIR3DL2	0.128115099	*	0.079987159	0.1382
	KIR3DL3	0.032494912	0.5327	0.023996555	0.6569
	KIR2DS4	0.099460245	0.0556	0.109239811	*
**Dendritic cell**	HLA-DPB1	0.505105784	****	0.417565933	****
	HLA-DQB1	0.340864367	****	0.233194409	****
	HLA-DRA	0.527044652	****	0.451615068	****
	HLA-DPA1	0.559072889	****	0.488731572	****
	CD1C	0.506846064	****	0.4110962	****
	NRP1	0.634177992	****	0.630304624	****
	ITGAX	0.518528824	****	0.445647167	****

*p<0.05, **p<0.01, ***p<0.001, ****p<0.0001.

We further utilized the GEPIA database to exploit the interaction between CD93 expression and well-known immune checkpoints in immunotherapy, such as PD-1, PD-L1, and CTLA-4 (**Figures 6C–E**). These findings support the apparent association of CD93 with immune infiltration of LIHC, which plays a key role in the immune response.

### 3.7 Prognostic evaluation of CD93 expression on the basis of immune cells in LIHC patients

**Figure 7:**
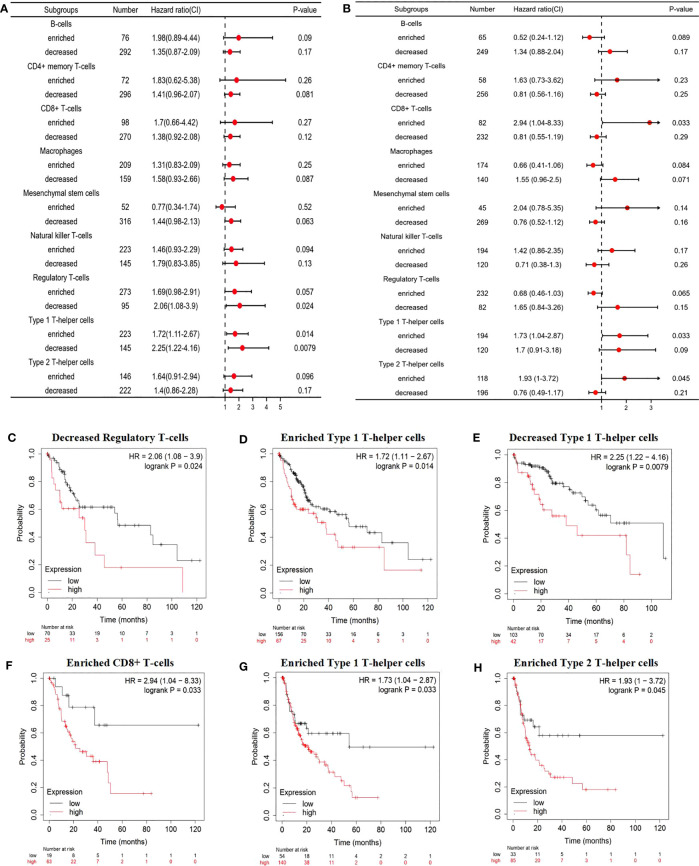
Prognostic evaluation of CD93 expression based on immune cells. A forest plot showing the correlations between **(A)** OS and **(B)** PFS and the CD93 expression according to different immune cell subgroups in LIHC patients. Correlations between CD93 expression and OS in **(C)** decreased Regulatory T-cells, **(D)** enriched Type 1 T-helper cells, **(E)** decreased Type 1 T-helper cells by Kaplan-Meier plotter; correlations between CD93 expression and PFS in **(F)** CD8+ T cells, **(G)** enriched Type 1 T-helper cells, and **(H)** enriched Type 2 T-helper cells.

With CD93 expression known to correlate with poor OS and PFS in LIHC, we further evaluated the impact of CD93 expression with the degree of infiltration of various immune cell subtypes on the prognosis of LIHC through prognostic analysis. The results of our analysis revealed a poor OS when LIHC patients with high expression of CD93 had decreased Regulatory T-cells, enriched Type 1 T-helper cells, and decreased Type 1 T-helper cells (**Figure 7A**). More details were shown in **Figures 7C–E**. In addition, LIHC patients with high expression of CD93 possessed poor PFS when there were enriched CD8+ T cells, enriched Type 1 T-helper cells, and enriched Type 2 T-helper cells (**Figures 7B, F–H**). These results suggest that CD93 affects the prognosis of patients with LIHC partially due to immune infiltration.

## 4 Discussion

LIHC is considered one of the most common malignancies with high morbidity and mortality worldwide (32, 33). To reduce the economic loss and life damage from LIHC, there emerge increasingly advanced and precise diagnoses and treatments. In particular, immunotherapy with tumor immune checkpoint inhibitors has revolutionized the treatment of many types of cancer, which brings the vast majority of patients too much real clinical benefit (34–37). However, LIHC is still diagnosed at an advanced stage and has a poor prognosis when it is found. Thus, it is urgently needed to clarify the mechanisms of hepatocarcinogenesis and identify useful prognostic biomarkers and potential immunotherapy targets of LIHC.

In this study, we found that CD93 could play an important role in LIHC by involving in endothelium development and angiogenesis. The researchers have reported that CD93 took part in the control of endothelial cell function through the cooperation between CD93 and dystroglycan, a laminin-binding protein, in malignant tumors (13, 38–40). CD93 overexpression was found in tumor vasculatures, and it influenced the survival of patients in PDAC, PNET, melanoma, and colon cancer (21, 41, 42). Our findings further demonstrated that CD93 was closely correlated with angiogenesis in LIHC, as among the most frequently altered genes closely associated with CD93 are many genes associated with tumor vascularization. In particular, IGFBP7 is a protein positively and significantly correlated with CD93 that has been identified to be up-regulated in tumor blood vessels and able to promote vascular angiogenesis. Hindering the CD93-IGFBP7 axis by CD93 or IGFBP7 mAb could normalize tumor vasculature to suppress tumor growth (21). Importantly, blocking the axis also increased immune cell infiltration to inhibit tumor progression (38).

Our study has shown that CD93 was positively correlated with six types of immune cells. There were a lot of studies that reported that CD93 was involved in the regulation of the immune response in different cancers (43–46). We also found that there was a close and tight interaction between CD93 expression and well-known immune checkpoints in immunotherapy, such as PD-1, PD-L1, and CTLA-4, which were extensively reported to play an immune escape role by PI3K/Akt signaling pathway or MAPK signaling pathway (47–50). CD93 may interact with them through these pathways. In addition, we explored the impact of the relationship between immune infiltration and CD93 expression on the prognosis and survival of patients. Furthermore, subgroup analysis by immune cells showed that high CD93 expression with enriched immune cells such as CD8+ T cells, Type 1 T-helper cells or Type 2 T-helper was highly related to poor prognosis in LIHC patients. Sun et al. observed that blocking the CD93 pathway can sensitize tumors to immunotherapy to promote the cancer immunotherapy effect and Riethe Huang et al. reported that CD93 could serve as an important regulator of leukemia stem cells and a potential therapeutic target (17, 20, 46, 51).

Above all these results, we can find that CD93 plays a role in LIHC through immune infiltration, and is expected to be a potential immunotherapy target.

## Data availability statement

The original contributions presented in the study are included in the article/**Supplementary Materials**. Further inquiries can be directed to the corresponding authors.

## Ethics statement

The studies involving human participants were reviewed and approved by the ethics committee of Shanghai General Hospita. The patients/participants provided their written informed consent to participate in this study.

## Author contributions

QJ, JK, and ZJ wrote this manuscript and prepared the table and the figure. LZ and WD devised and supervised this project. WQ, PW, and WH assisted in the literature search and manuscript editing. All authors contributed to the article and approved the submitted version.
