# Supplementary figures and images for: CD93 overexpresses in liver hepatocellular carcinoma and represents a potential immunotherapy target
Source: Front Immunol. 2023 Jul 7;14:1158360. doi: 10.3389/fimmu.2023.1158360 (PMC10359974; doi:10.3389/fimmu.2023.1158360)

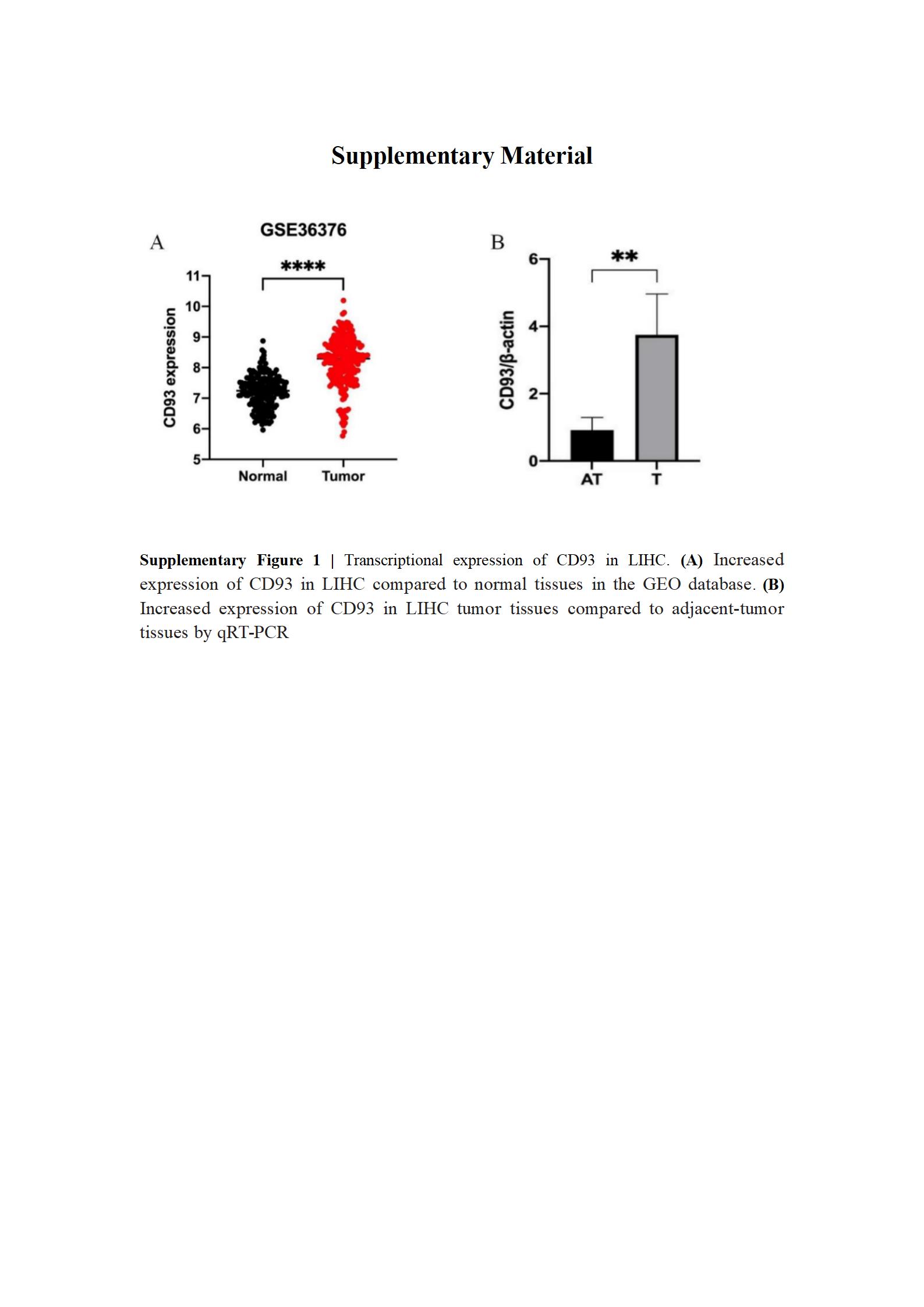

Supplement: Supplementary file 1 [file Image_1.jpg]

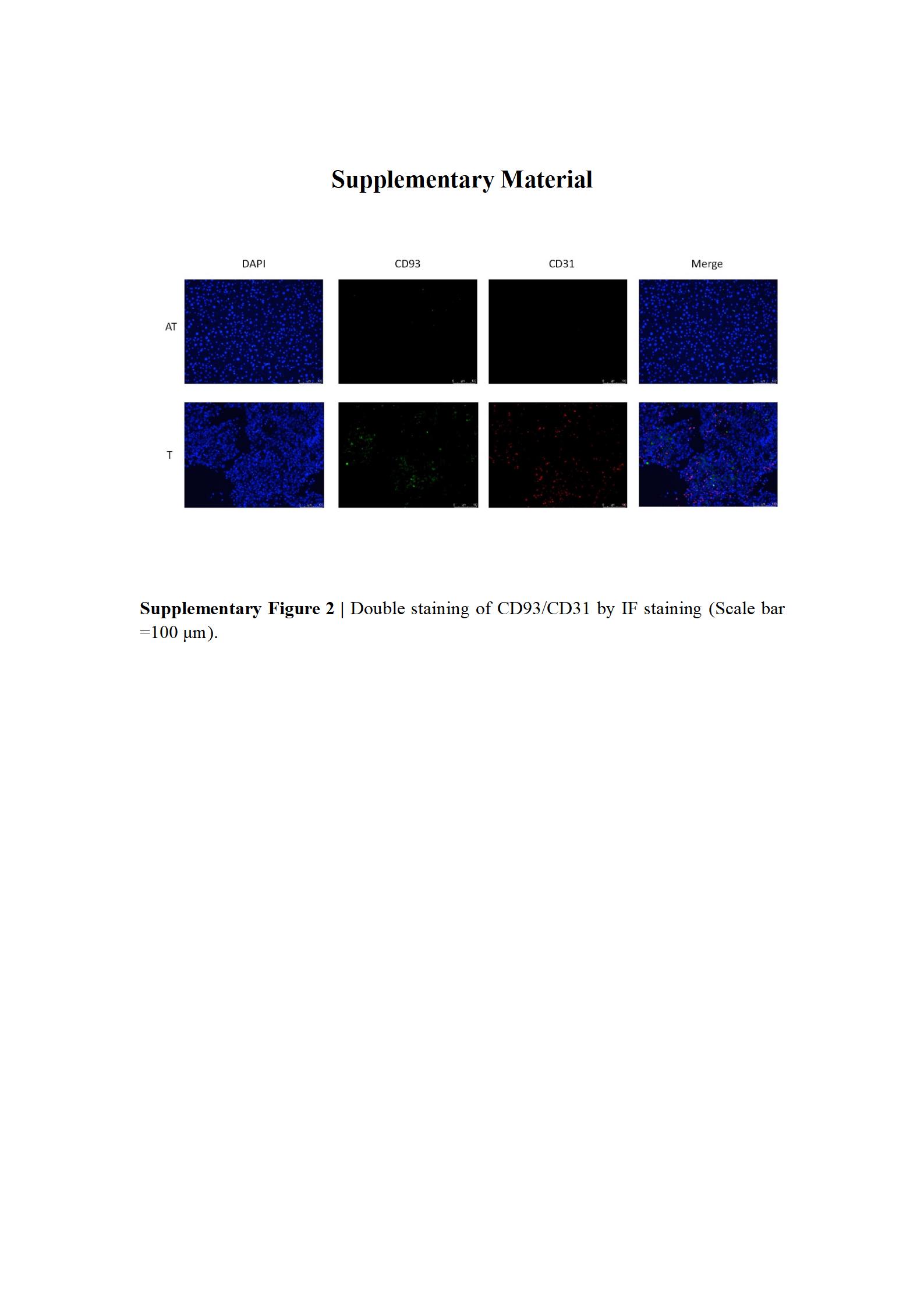

Supplement: Supplementary file 2 [file Image_2.jpg]

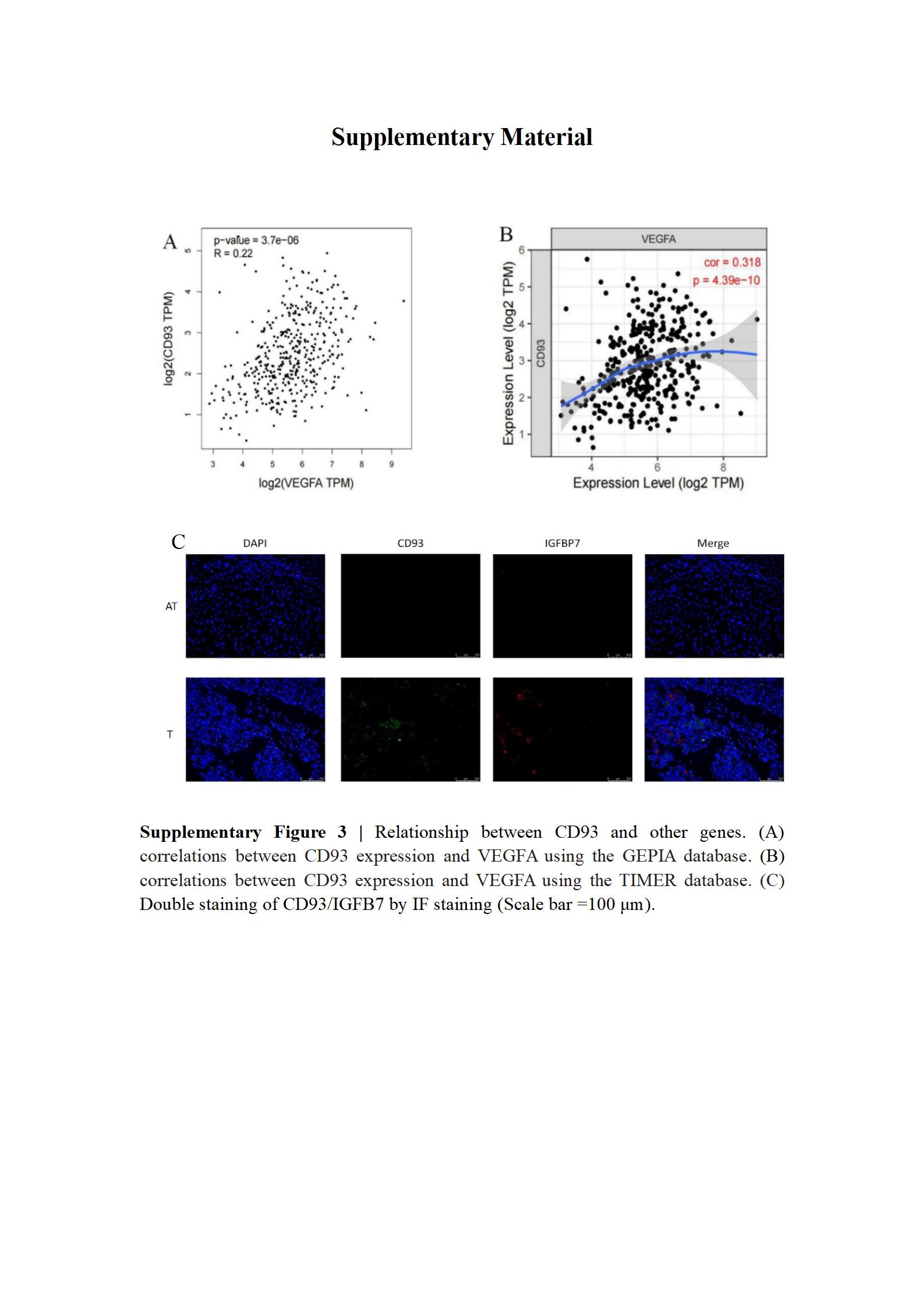

Supplement: Supplementary file 3 [file Image_3.jpg]
